# Multi-biobank Mendelian randomization analyses identify opposing pathways in plasma low-density lipoprotein-cholesterol lowering and gallstone disease

**DOI:** 10.1007/s10654-024-01141-5

**Published:** 2024-07-15

**Authors:** Guoyi Yang, Amy M Mason, Dipender Gill, C Mary Schooling, Stephen Burgess

**Affiliations:** a School of Public Health, Li Ka Shing Faculty of Medicine, The University of Hong Kong, Hong Kong, China; b MRC Biostatistics Unit, University of Cambridge, Cambridge, UK; c British Heart Foundation Cardiovascular Epidemiology Unit, Department of Public Health and Primary Care, University of Cambridge, Cambridge, UK; d Victor Phillip Dahdaleh Heart and Lung Research Institute, University of Cambridge, Cambridge, UK; e Department of Epidemiology and Biostatistics, School of Public Health, Imperial College London, London, UK; f Graduate School of Public Health and Health Policy, City University of New York, New York, United States

**Keywords:** LDL-cholesterol, gallstone disease, drug-target Mendelian randomization, clustered Mendelian randomization

## Abstract

**Background:**

Plasma low-density lipoprotein (LDL)-cholesterol is positively associated with coronary artery disease risk while biliary cholesterol promotes gallstone formation. Different plasma LDL-cholesterol lowering pathways may have distinct effects on biliary cholesterol and thereby gallstone disease risk.

**Methods:**

We conducted a Mendelian randomization (MR) study using data from the UK Biobank (30,547 gallstone disease cases/336,742 controls), FinnGen (34,461 cases/301,383 controls) and Biobank Japan (9,305 cases/168,253 controls). We first performed drug-target MR analyses substantiated by colocalization to investigate the effects of plasma LDL-cholesterol lowering therapies on gallstone disease risk. We then performed clustered MR analyses and pathway analyses to identify distinct mechanisms underlying the association of plasma LDL-cholesterol with gallstone disease risk.

**Results:**

For a 1-standard deviation reduction in plasma LDL-cholesterol, genetic mimics of statins were associated with lower gallstone disease risk (odds ratio 0.72 [95% confidence interval 0.62, 0.83]), but genetic mimics of PCSK9 inhibitors and targeting apolipoprotein B were associated with higher risk (1.11 [1.03, 1.19] and 1.23 [1.13, 1.35]). The association for statins was supported by colocalization (posterior probability 98.7%). Clustered MR analyses identified variant clusters showing opposing associations of plasma LDL-cholesterol with gallstone disease risk, with some evidence for ancestry-and sex-specific associations. Among variants lowering plasma LDL-cholesterol, those associated with lower gallstone disease risk were mapped to glycosphingolipid biosynthesis pathway, while those associated with higher risk were mapped to pathways relating to plasma lipoprotein assembly, remodelling, and clearance and ATP-binding cassette transporters.

**Conclusions:**

Different plasma LDL-cholesterol lowering pathways have distinct and opposing effects on risk of gallstone disease.

## Introduction

Plasma low-density lipoprotein (LDL)-cholesterol is positively associated with coronary artery disease (CAD) risk [1], whereas biliary cholesterol promotes cholesterol gallstones formation [2]. A recent randomized controlled trial (RCT) showed lowering plasma LDL-cholesterol with bempedoic acid, an adenosine triphosphate (ATP) citrate lyase inhibitor, increased risk of gallstones [3], but trial evidence for other lipid modifiers is limited [4]. Therefore, it remains unclear whether the lithogenic effect is a general consequence of lowering plasma LDL-cholesterol or is unique to bempedoic acid.

RCTs are not usually designed or powered to identify adverse effects or novel indications. Observational studies suggest statin use is associated with lower gallstone disease risk [5, 6], but these studies could be biased due to residual confounding or selection bias. Mendelian randomization (MR), an instrumental variable analysis with genetic instruments, is more robust to confounding than conventional observational studies [7]. However, previous MR studies have yielded contradictory results, suggesting a positive [8, 9] or null [10] association of lower plasma LDL-cholesterol with gallstone disease risk. Genetic studies of specific gene regions did not show a uniform association of lower plasma LDL-cholesterol with gallstone disease risk [9–13]. Nevertheless, the possibility of systematic differences in the lithogenic effects of different plasma LDL-cholesterol lowering pathways has less often been considered.

Plasma LDL-cholesterol is regulated by different biological pathways [14], which could have distinct effects on biliary cholesterol and thereby gallstone disease. For example, statins reduce plasma LDL-cholesterol by inhibiting cholesterol biosynthesis [15], which may decrease biliary cholesterol [16] and reduce gallstone disease risk. However, pathways reducing plasma LDL-cholesterol while elevating biliary cholesterol, such as activating adenosine triphosphate (ATP)-binding cassette transporters G5/8 (ABCG5/8), may increase gallstone disease risk [11].

We hypothesized that different plasma LDL-cholesterol lowering pathways have distinct effects on risk of gallstone disease. First, we used MR to assess the associations of genetic mimics of current and emerging plasma LDL-cholesterol lowering therapies with gallstone disease risk. Second, we investigated distinct pathways underlying the association of plasma LDL-cholesterol with gallstone disease risk. Where possible, we assessed ancestry- and sex-specific associations, because gallstone prevalence is higher in European than Asian ancestry individuals, and in women than men [2].

## Methods

### Study design

We used individual-level data from UK Biobank [17] and summary-level data from FinnGen [18] and Biobank Japan [19] for gallstone disease. We selected genetic mimics of plasma LDL-cholesterol lowering therapies from genes encoding the molecular target of each therapy. We conducted drug-target MR analyses to assess the associations of genetic mimics of each therapy with gallstone disease risk. We performed colocalization analyses to examine whether any associations found were driven by a shared causal variant between exposure and outcome or were confounded by linkage disequilibrium [20].

We extracted genetic predictors for plasma LDL-cholesterol from across the genome. We conducted clustered MR analyses to identify distinct clusters of genetic variants having similar causal estimates for plasma LDL-cholesterol on gallstone disease risk [21]. We performed pathway analyses to investigate biological pathways relating to each cluster. A summary of the study design is shown in eFig.1 (Online resource).

### Data sources

The UK Biobank recruited approximately 500,000 people (intended age 40-69 years, 94% self-reported European ancestry) between 2006 and 2010 from across the United Kingdom [17]. Individual-level data used were under application 98032 (October 2021 updated). Cases were defined based on self-reported history of gallstones, International Classification of Diseases (ICD)-9 and ICD-10 codes related to gallstones, and medical treatment of gallstones (Online resource: eTable 1), as previously [22]. Both prevalent and incident cases were included. Controls were individuals without gallstone-related disease or treatment. Individuals who underwent cholecystectomy due to an alternative pathology (e.g., neoplasm) were excluded. We included 367,289 unrelated individuals of European ancestry (30,547 gallstone disease cases and 336,742 controls) with genomic data passing quality control as described previously [23]. We used logistic regression to obtain sex-combined and sex-specific genetic associations with gallstone disease.

Summary-level data from FinnGen (R8 release) included 34,461 gallstone cases and 301,383 controls (mean age 52 years, 55.7% women) [18]. Cases were defined based on ICD-8, 9, 10 codes [18]. Summary-level data from Biobank Japan included 9,305 gallstone cases and 168,253 controls (mean age 63 years, 46.3% women) [19]. Cases were defined based on ICD-10 codes [19]. Definitive codes of gallstone disease are provided in eTable 1 (Online resource).

### Genetic instruments

We selected genetic instruments based on their associations with plasma LDL-cholesterol obtained from ancestry-specific summary-level data from the Global Lipids Genetics Consortium (GLGC) (1,231,289/82,587 people of European/East Asian ancestry) [24], but not based on functional information. We selected genetic mimics of plasma LDL-cholesterol lowering therapies from genes encoding the molecular targets of each therapy (i.e., *HMGCR* for statins, *PCSK9* for proprotein convertase subtilisin/kexin type 9 (PCSK9) inhibitors, *NPC1L1* for ezetimibe, *ACLY* for ATP citrate lyase inhibitors, *LDLR* for targeting LDL receptors, and *APOB* for targeting apolipoprotein B (apoB)), as previously [25, 26]. We used genetic mimics of targeting ABCG5/8 from *ABCG5/8* as a positive control exposure, because *ABCG5/8* is a well-established lithogenic gene [2]. We included all variants within 100kb on either side of each target gene that were in low linkage disequilibrium (r^2^ <0.1) and were genome-wide significantly (*p* value <5×10^-8^) associated with plasma LDL-cholesterol. We used a less stringent cut-off for linkage disequilibrium (r^2^ <0.1) to obtain more variants in each gene region to increase the power [27]. We also extracted genetic predictors for plasma LDL-cholesterol from across the genome that were uncorrelated (r^2^ <0.001) and genome-wide significantly (*p* value <5×10^-8^) associated with plasma LDL-cholesterol. Estimates were expressed in 1-standard deviation (around 0.87 mmol/L) reduction in plasma LDL-cholesterol.

We used the F-statistic to assess instrument strength, approximated by the square of each SNP-exposure association divided by the square of its standard error [28]. We used PhenoScanner, a database of genotype-phenotype associations [29, 30], to check whether SNPs were genome-wide significantly (*p* value <5×10^-8^) associated with common confounders (i.e., socioeconomic status, smoking, alcohol drinking and physical activity). We included these SNPs in the main analysis, and excluded them in the sensitivity analysis. We also used positive control outcomes, i.e., CAD from CARDIoGRAMplusC4D Consortium (60,801 cases and 123,504 controls) [31] for people of European ancestry and myocardial infarction (MI) from Biobank Japan (14,992 cases and 146,214 controls) [19] for East Asians. A summary of genome-wide association studies (GWAS) used is provided in eTable 2 (Online resource).

### MR analysis

We aligned SNPs based on alleles and allele frequencies. We used proxy SNPs (r^2^≥0.8), where possible, when SNPs were not available in the outcome GWAS. We used the 1000 Genomes reference panel to obtain linkage disequilibrium. We selected a European population for proxy SNPs in the UK Biobank and FinnGen, and an East Asian population for proxy SNPs in Biobank Japan. We calculated MR estimates by meta-analyzing Wald estimates (the ratio of the genetic association with outcome to the genetic association with exposure) using inverse variance weighting (IVW) with fixed effects for three SNPs or fewer and random effects for four SNPs or more [32]. To assess the robustness of the IVW estimates, we conducted sensitivity analyses using weighted median [33] and MR Egger [34]. We used the MR Egger intercept to assess directional pleiotropy [34]. For uncorrelated SNPs (r^2^ <0.001), we used IVW [32] and MR Egger [34] assuming independent genetic instruments. For the SNPs in low linkage disequilibrium (r^2^ <0.1), we used IVW [27] and MR Egger [35] extended to account for correlations between genetic instruments by fitting a generalized weighted linear regression model using a weighting matrix._

We meta-analyzed MR estimates from the three biobanks using a fixed-effects model unless the Q-statistic suggested heterogeneity when we used a random-effects model. We assessed differences by sex using a two-sided z-test [36].

### Colocalization analysis

We performed colocalization analyses in a Bayesian framework to assess whether any associations found in drug-target MR were driven by a shared causal variant between plasma LDL-cholesterol and gallstone disease or were confounded by linkage disequilibrium [20]. This method uses Approximate Bayes Factor computations to assess the posterior probability of several hypotheses [20]. H_0_, no association with either trait; H_1_, association with plasma LDL-cholesterol only; H_2_, association with gallstone disease only; H_3_, associations of two independent variants and one for each trait; H_4_, associations of one shared variant with both traits [20]. A probability of H_4_ larger than 0.80 provides evidence for colocalization [20]. We included variants (minor allele frequency >0.1%) in or near (+/-100kb) the target gene where any associations were identified. We set the prior probabilities as recommended, i.e., 1.0e-4 for a variant associated with plasma LDL-cholesterol, 1.0e-4 for a variant associated with gallstone disease, and 1.0e-5 for a variant associated with both traits [20]. We also calculated the posterior probability for a shared variant associated with both traits conditional on the presence of a variant associated with gallstone disease, as the power to detect colocalization is low when the variants are not strongly associated with the outcome [37].

### Clustered MR

We used the MR-Clust method to identify distinct clusters of genetic variants having similar causal estimates for plasma LDL-cholesterol on gallstone disease, which might reflect distinct biological pathways [21]. The MR-Clust accounts for differential uncertainty in the causal estimates, and includes a null cluster where SNP-specific estimates are centred around zero and a junk cluster where SNP-specific estimates are highly dispersed and are considered outliers [21]. The presence of null and junk clusters requires substantial evidence of similarity to define a cluster, which avoids the detection of spurious clusters [21]. We only included variants with inclusion probability >0.80 in each cluster, and only reported a cluster if at least four variants satisfy this criterion, as recommended [21].

### Pathway analysis

We performed pathway analysis to examine biological pathways relating to each variant cluster using the Functional Mapping and Annotation (FUMA) platform, which includes SNP2GENE and GENE2FUNC functions [38]. We first applied the SNP2GENE function to map cluster-specific genetic variants to genes, where we used 100-kb positional mapping and expression quantitative trait locus (eQTL) mapping based on GTEx v8 [38]. We then used the GENE2FUNC function to associate the mapped genes with biological pathways defined by KEGG and Reactome database [38].

All statistical analyses were conducted using R version 4.2.1 and the packages “ieugwasr”, “TwoSampleMR”, “MendelianRandomization”, “metafor”, “coloc”, and “mrclust”.

## Results

### Baseline characteristics of UK Biobank participants

Baseline characteristics of 367,289 UK Biobank participants (cases = 21,201 women/9,346 men, controls = 177,478 women/159,264 men) included in this study are shown in Table 1. Cases were older at baseline (mean age 59.4 vs 57.0 years) and had higher body mass index (mean 29.6 vs 27.2 kg/m^2^) than controls. Cases had a greater proportion of women (69.4% vs 52.7%) and current lipid-lowering medication users (22.1% vs 16.5%) but had a smaller proportion of current alcohol drinkers (89.1% vs 93.6%) than controls.

### Genetic instruments

We extracted SNPs for plasma LDL-cholesterol lowering therapies in people of European and East Asian ancestry, respectively (Online resource: eTable 3). We were unable to extract SNPs for ATP citrate lyase inhibitors in people of European or East Asian ancestry, or for ezetimibe and targeting ABCG5/8 in East Asians, because the SNPs in or near the target genes were not genome-wide significantly associated with plasma LDL-cholesterol (*p* values >5×10^-8^). We extracted 324 and 43 SNPs for plasma LDL-cholesterol in people of European and East Asian ancestry, respectively (Online resource: eTable 4).

The F-statistics for all SNPs were >10. Variance in plasma LDL-cholesterol explained by SNPs for each therapy and plasma LDL-cholesterol is provided in eTable 5 (Online resource). None of the SNPs for each therapy were genome-wide significantly associated with common confounders (*p* values >5×10^-8^), but four SNPs for plasma LDL-cholesterol were associated with smoking or alcohol drinking (Online resource: eTable 6). We included these four SNPs in the main analysis and excluded them in the sensitivity analysis. As expected, SNPs for each therapy and lower plasma LDL-cholesterol were associated with lower risk of CAD and MI (Online resource: eFig.2-3).

### Drug-target MR

**Fig. 1:**
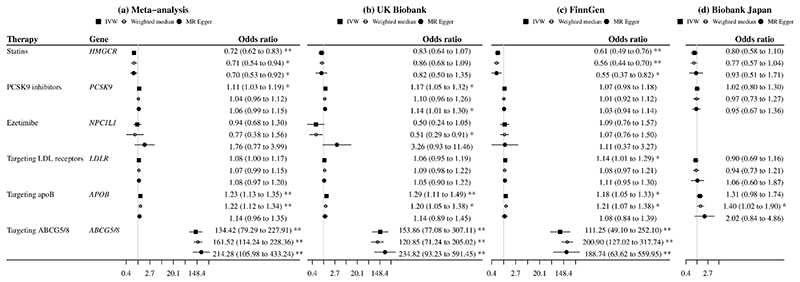
Mendelian randomization estimates for genetic mimics of plasma LDL-cholesterol lowering therapies on risk of gallstone disease ABCG5/8, adenosine triphosphate (ATP)-binding cassette transporters G5/8; ApoB, apolipoprotein B; IVW, inverse variance weighted; LDL, low-density lipoprotein; PCSK9, proprotein convertase subtilisin/kexin type 9. Estimates are expressed in odds ratio per 1-standard deviation (around 0.87 mmol/L) reduction in plasma LDL-cholesterol. * denotes *p* value <0.05; ** denotes *p* value <0.001. *P* values (MR Egger intercept) for statins, PCSK9 inhibitors, ezetimibe, targeting LDL receptors, targeting apoB, and targeting ABCG5/8 were 0.98, 0.33, 0.002, 0.81, 0.21, and 0.18 in the UK Biobank, 0.53, 0.03, 0.98, 0.60, 0.43, and 0.16 in FinnGen, and 0.57, 0.60, NA, 0.52, 0.31, and NA in Biobank Japan.

After meta-analyzing MR estimates from the three biobanks, genetic mimics of statins were associated with lower gallstone disease risk, while genetic mimics of PCSK9 inhibitors, targeting apoB, and targeting ABCG5/8 were associated with higher gallstone disease risk (Fig.1). Targeting LDL receptors had a similar IVW estimate as PCSK9 inhibitors, despite the *p* value of 0.06 (Fig.1). We did not observe heterogeneity in IVW estimates across biobanks (*p* values for heterogeneity >0.05). The weighted median and MR Egger gave similar interpretations, although the MR Egger intercept indicated possible directional pleiotropy for ezetimibe (UK Biobank) and PCSK9 inhibitors (FinnGen) (Fig.1). For a 1-standard deviation reduction in plasma LDL-cholesterol, associations for statins (odds ratio for women 0.76 [95% confidence interval 0.58, 1.00] vs men 0.99 [0.62 to 1.58]), PCSK9 inhibitors (women 1.09 [0.95 to 1.26] vs men 1.36 [1.11 to 1.67]) and targeting ABCG5/8 (women 243.40 [114.10 to 519.26] vs men 58.39 [29.94 to 113.86]) slightly differed by sex (Online resource: eFig.4, *p* values for sex differences 0.332, 0.081 and 0.006).

### Colocalization analysis

**Fig. 2:**
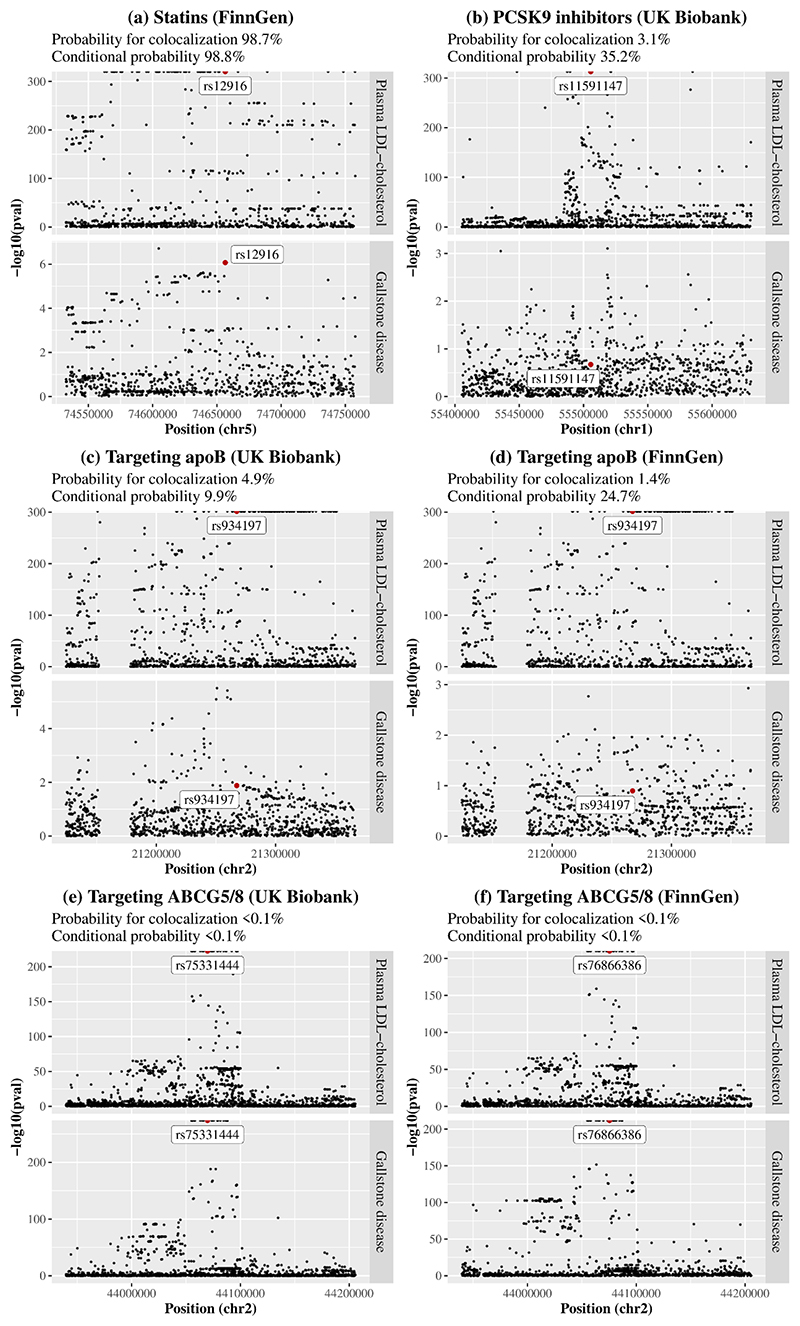
Colocalization analyses for plasma LDL-cholesterol with gallstone disease in or near (+-100kb) the target gene of each therapy

Colocalization analyses were performed for plasma LDL-cholesterol with gallstone disease in or near (+-100kb) *HMGCR, PCSK9, APOB*, and *ABCG5/8* using the UK Biobank and FinnGen. The posterior probability for a shared variant associated with both traits was 98.7% for the *HMGCR* gene region but <10% for the other gene regions (Fig.2). At the *HMGCR* gene region, conditional on the existence of a shared variant associated with both traits, rs12916 was the shared variant with posterior probability >99.9%. The posterior probability for a variant associated with plasma LDL-cholesterol only was >50% for the *PCSK9* and *APOB* gene regions; the posterior probability for independent variants associated with each trait was > 99.9% for the *ABCG5/8* gene region (Online resource: eTable 7).

ABCG5/8, adenosine triphosphate (ATP)-binding cassette transporters G5/8; ApoB, apolipoprotein B; LDL, low-density lipoprotein; PCSK9, proprotein convertase subtilisin/kexin type 9. Prior probabilities were set to 1.0e-4 for a variant associated with plasma LDL-cholesterol, 1.0e-4 for a variant associated with gallstone disease, and 1.0e-5 for a variant associated with both traits. Probability for colocalization means the posterior probability for a shared variant associated with both traits; conditional probability means the posterior probability for a shared variant associated with both traits conditional on the presence of a variant associated with gallstone disease. The variant with the largest posterior probability for both traits is highlighted with a label.

### Clustered MR

**Fig. 3:**
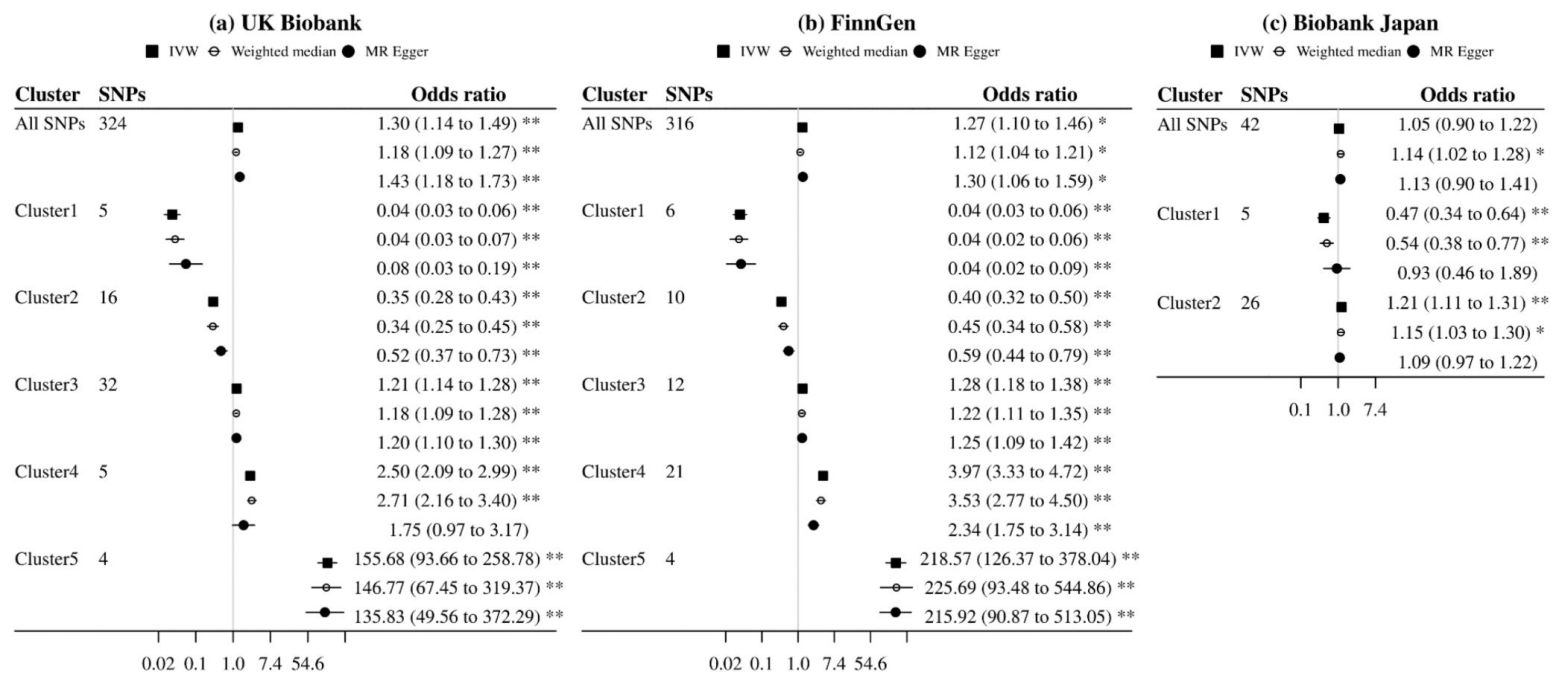
Mendelian randomization estimates for genetically predicted lower plasma LDL-cholesterol on risk of gallstone disease using all SNPs and cluster-specific SNPs (inclusion probability >0.80) IVW, inverse variance weighted; LDL, low-density lipoprotein. Estimates are expressed in odds ratio per 1-standard deviation (around 0.87 mmol/L) reduction in plasma LDL-cholesterol. * denotes *p* value <0.05; ** denotes *p* value <0.001. *P* values (MR Egger intercept) for all SNPs, cluster 1, cluster 2, cluster 3, cluster 4, and cluster 5 were 0.19, 0.07, 0.01, 0.78, 0.22, and 0.76 in the UK Biobank, 0.70, 0.88, 0.001, 0.65, <0.001, and 0.97 in FinnGen, and 0.38, 0.04, 0.01, NA, NA, and NA in Biobank Japan.

**Fig. 4:**
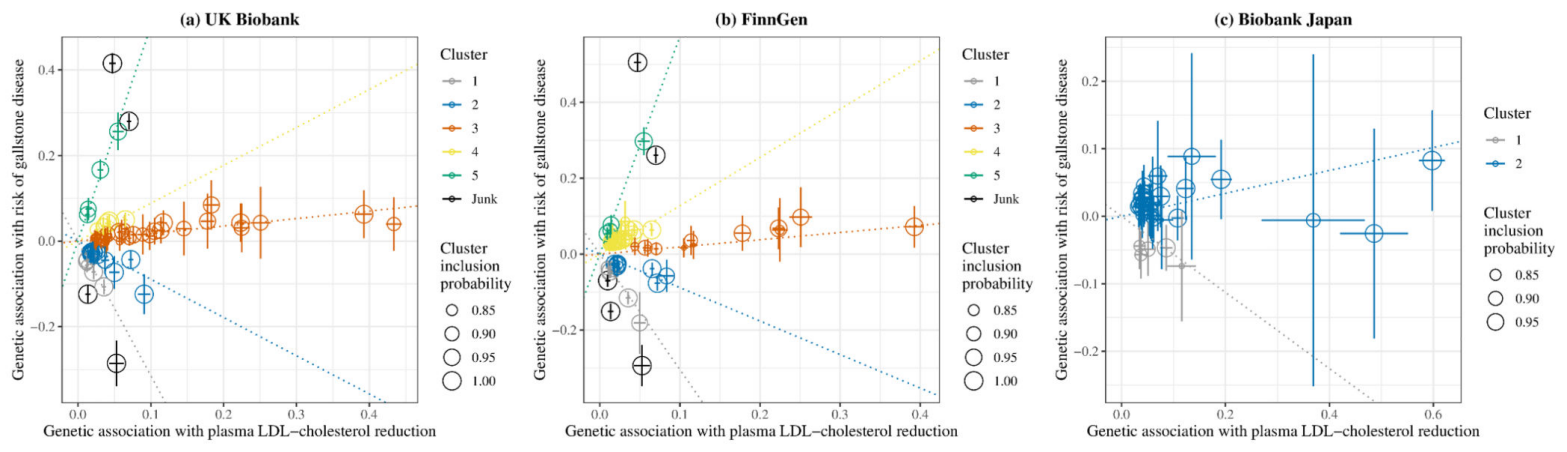
Genetic associations with plasma LDL-cholesterol reduction (standard deviation) and risk of gallstone disease (log odds) for SNPs with inclusion probability >0.80 in clustered Mendelian randomization analyses LDL, low-density lipoprotein. Points represent SNPs; dotted lines are cluster means; error bars are 95% confidence intervals for genetic associations. A positive slope indicates an association of lower plasma LDL-cholesterol with higher risk of gallstone disease; a negative slope indicates an association of lower plasma LDL-cholesterol with lower risk of gallstone disease.

Using all SNPs for lower plasma LDL-cholesterol, there was a positive association with gallstone disease risk in the UK Biobank and FinnGen but a null association in Biobank Japan (Fig.3). However, we observed heterogeneity in SNP-specific estimates (*p* values for heterogeneity <0.001). Clustered MR analyses consistently identified variant clusters showing opposing associations of lower plasma LDL-cholesterol with gallstone disease risk (Fig.3-4). The MR Egger intercept indicated possible directional pleiotropy for cluster 2 (UK Biobank), cluster 2 and 4 (FinnGen), and cluster 1 and 2 (Biobank Japan); however, the weighted median and MR Egger estimates were in the same direction as the IVW estimates (Fig.3). By contrast, these clusters were generally associated with lower risk of CAD and MI (Online resource: eFig.3). Sensitivity analysis excluding SNPs associated with smoking or alcohol drinking did not change the results substantially (Online resource: eFig.5-6).

Cluster patterns were consistent in the UK Biobank and FinnGen (Fig.3-4), with overlapping cluster-specific SNPs (Online resource: eTable 8) and similar SNP-specific estimates (Online resource: eFig.7). Sex-specific clustered MR analyses also showed such opposing associations, but the pattern appeared more evident in women than men (Online resource: eFig.8-9).

### Pathway analysis

We found clusters 2 and 3 in FinnGen, cluster 3 in the UK Biobank, and cluster 2 in Biobank Japan mapped to specific pathways (Online resource: eTable 9). Cluster 2 in FinnGen showing an inverse association of lower plasma LDL-cholesterol with gallstone disease was mapped to glycosphingolipid biosynthesis pathway, while other clusters showing a positive association were mapped to pathways relating to plasma lipoprotein assembly, remodelling, and clearance and ATP-binding cassette transporters.

## Discussion

This trans-biobank MR study including 880,691 individuals (74,313 gallstone disease cases) provides genetic evidence that statins may reduce gallstone disease risk, consistent with previous observational studies [5, 6]. Our investigation has added to the evidence base by identifying distinct and opposing pathways underlying the association of plasma LDL-cholesterol with gallstone disease.

Genetic evidence suggested statins may reduce the risk of gallstone disease, while PCSK9 inhibitors, targeting apoB and targeting ABCG5/8 may increase the risk. These findings are consistent with observational studies showing long-term use of statins is associated with lower gallstone disease risk [5, 6], and a previous MR study showing *ABCG5/8* variants lowering plasma LDL-cholesterol are associated with higher gallstone disease risk [11]. However, an RCT including 9,270 individuals with chronic kidney disease showed simvastatin plus ezetimibe did not affect gallstone risk during a follow-up of 4.9 years, when 216 individuals developed gallstones [4]. A previous genetic study including 63,051 individuals (3,323 with symptomatic gallstones) showed variants in *PCSK9* or *APOB* were not associated with risk of symptomatic gallstone disease [10]. The small number of gallstone cases may have limited the detection of any possible effect in these studies. Meta-analyses of 21 RCTs showed statins reduced the risk of pancreatitis, a common complication of gallstone disease [39].

Plasma LDL-cholesterol lowering therapies may have distinct effects on biliary cholesterol and thereby gallstone disease. Statins decrease hepatic cholesterol synthesis [15], and may decrease biliary cholesterol [16] and facilitate cholesterol gallstone dissolution [40, 41]. PCSK9 inhibitors increase LDL receptors and targeting apoB decreases apoB-containing particles [15], which may increase hepatic and biliary cholesterol [2]. Activating ABCG5/8 facilitates cholesterol efflux into the intestine and the gallbladder, which increases biliary cholesterol [2]. Increased biliary cholesterol accelerates supersaturation of bile and promotes cholesterol gallstone formation [2].

Colocalization analysis substantiated the association of statins with gallstone disease. A lack of colocalization for PCSK9 inhibitors and targeting apoB is possibly due to insufficient power. However, colocalization analysis suggested the association of targeting ABCG5/8 with gallstone disease was confounded by linkage disequilibrium. This could be explained by different lead variants for plasma versus biliary cholesterol in or near *ABCG5/8*. ABCG5/8 increases cholesterol excretion into the intestine and the gallbladder and may have a larger effect on biliary cholesterol than plasma LDL-cholesterol. Such differences would also explain the implausibly high MR estimates for targeting ABCG5/8, which are presented in effect sizes of plasma LDL-cholesterol reduction.

Using all SNPs for lower plasma LDL-cholesterol, there was a positive or null association with gallstone disease, as shown in previous MR studies [8, 10]. However, we identified variant clusters showing opposing associations of plasma LDL-cholesterol with gallstone disease. Among variants predicting lower plasma LDL-cholesterol, those associated with lower gallstone disease risk were mapped to glycosphingolipid biosynthesis pathway, while those associated with higher gallstone disease risk were mapped to pathways relating to plasma lipoprotein assembly, remodelling, and clearance and ATP-binding cassette transporters. These findings are consistent with the evidence available and the mechanisms of plasma LDL-cholesterol lowering therapies. In vitro studies have showed statins affect glycosphingolipid profiles through inhibiting Rab prenylation [42], which could suppress gallstone formation [43]. PCSK9 inhibitors and targeting apoB are involved in plasma LDL assembly and clearance, and ABCG5/8 are key members of ATP-binding cassette transporters [15]. Correspondingly, targeting apoB had similar MR estimates as cluster 3 in FinnGen and UK Biobank and cluster 2 in Biobank Japan; targeting ABCG5/8 had similar MR estimates as cluster 5 in FinnGen and UK Biobank (Fig.1 and 3).

Unlike the atheroprotective effects, the lithogenic effects vary between different plasma LDL-cholesterol lowering pathways, which cast doubt on a causal role of plasma LDL-cholesterol in gallstone disease. Similarly, previous MR studies showed plasma LDL-cholesterol lowering therapies differed in their associations with body mass index [44] and type 2 diabetes [45]. These insights highlight the importance of pathway-specific investigations to identify novel indications for biomarkers, here specifically plasma LDL-cholesterol.

The opposing associations of plasma LDL-cholesterol with gallstone disease seemed more evident in European than East Asian ancestry individuals and in women than men, consistent with different gallstone prevalence rates by ancestry and sex [2]. Plasma LDL-cholesterol lowering therapies may have distinct effects on biliary cholesterol and thereby cholesterol gallstones; however, pigment gallstones are more common in East Asians than Europeans [46], which may explain the difference by ancestry. Statins partially operate through sex hormones [47], which could be relevant to the more marked association in women than men. Alternatively, statins decreasing calcium, another component of gallstones, specifically in women [48] might play a role.

This trans-ancestry study takes advantage of three large-scale biobanks. A systematic difference in the lithogenic effects of different plasma LDL-cholesterol lowering pathways is novel. This study has several limitations. First, MR relies on three rigorous assumptions, that is genetic instruments should be strongly related to the exposure, share no common cause with the outcome, and be independent of the outcome given the exposure [7]. We calculated the F-statistics, checked genetic associations with common confounders, and used CAD and MI as positive control outcomes to assess the validity of genetic instruments. Second, we were unable to assess the lithogenic effect of ATP citrate lyase inhibitors due to a lack of valid instruments. Although variants in the *ACLY* gene have been used in a prior Mendelian randomization analysis, none of these variants were associated with plasma LDL-cholesterol at even a suggestive level of genome-wide significance (*p* values >10^-3^ for all variants) [26]. Third, we performed colocalization analyses for plasma LDL-cholesterol with gallstone disease, while biliary cholesterol likely underlies any effects on gallstone disease. The discrepancy between plasma versus biliary cholesterol may partly explain some lack of colocalization. Replication using biliary cholesterol would be ideal when relevant GWAS becomes available. Fourth, not all the variant clusters could be mapped to specific pathways, possibly due to the small number of SNPs with inclusion probability >0.80 in some clusters. However, such a conservative inclusion criterion avoids the generation of spurious clusters by chance [21]. Fifth, meta-analysis of MR estimates across the biobanks can be difficult to interpret, as they included individuals of different ancestries and can be open to different sources of bias. Gallstone disease studied here is not specific to cholesterol gallstones and the definitions slightly vary across the three biobanks; however, we still observed consistent patterns. Sixth, we did not take into account potential non-linearity, because a previous MR study suggested a linear association of plasma LDL-cholesterol with gallstone disease risk [9]. Finally, MR assesses lifelong effects, which cannot directly inform the quantitative effects of plasma LDL-cholesterol lowering therapies in the short term.

## Conclusions

This genetic study supports that different plasma LDL-cholesterol lowering pathways have opposing effects on risk of gallstone disease. Specifically, statins may decrease the risk of gallstone disease but PCSK9 inhibitors and targeting apoB may increase the risk. Plasma LDL-cholesterol lowering through glycosphingolipid biosynthesis pathway may decrease the risk of gallstone disease but through pathways relating to plasma lipoprotein assembly, remodelling, and clearance and ATP-binding cassette transporters may increase the risk.

## Supplementary Material

Supplementary material

## Figures and Tables

**Table 1:** Baseline characteristics of gallstone disease cases and controls in the UK Biobank

Characteristics	Cases (*N*=30,547)	Controls (*N*=336,742)
Age at recruitment, years	59.4 (7.4)	57.0 (8.0)
Sex		
Men	9,346 (30.6)	159,264 (47.3)
Women	21,201 (69.4)	177,478 (52.7)
LDL-cholesterol, mmol/L	3.5 (0.9)	3.6 (0.9)
BMI, kg/m^2^	29.6 (5.5)	27.2 (4.6)
SBP, mmHg	138.8 (18.4)	137.6 (18.6)
Smoking		
Current	3,118 (10.2)	34,712 (10.3)
Other	27,429 (89.8)	302,030 (89.7)
Alcohol drinking		
Current	27,204 (89.1)	315,226 (93.6)
Other	3,343 (10.9)	21,516 (6.4)
Lipid-lowering medication		
Current	6,751 (22.1)	55,467 (16.5)
Other	23,796 (77.9)	281,275 (83.5)

BMI, body mass index; LDL, low-density lipoprotein; SBP, systolic blood pressure. Data are mean (standard deviation) for continuous variables or *N* (%) for categorical variables.

## Data Availability

This study has been conducted using the UK Biobank Resource under Application number 98032. Summary-level data analyzed are available in the website http://csg.sph.umich.edu/willer/public/glgc-lipids2021/ for GLGC, https://www.finngen.fi/en/access_results for FinnGen, https://pheweb.jp/downloads for Biobank Japan, and http://www.cardiogramplusc4d.org/data-downloads/ for CARDIoGRAMplusC4D Consortium. The R code for data analysis is shared in the GitHub https://github.com/YANGGYEMMA/LDL_and_gallstones.

## Abbreviations

ABCG5/8: adenosine triphosphate-binding cassette transporters G5/8
ApoB: apolipoprotein B
ATP: adenosine triphosphate
CAD: coronary artery disease
eQTL: expression quantitative trait locus
FUMA: Functional Mapping and Annotation
GLGC: Global Lipids Genetics Consortium
GWAS: genome-wide association studies
ICD: International Classification of Diseases
IVW: inverse variance weighting
LDL: low-density lipoprotein
MI: myocardial infarction
MR: Mendelian randomization
PCSK9: proprotein convertase subtilisin/kexin type 9
RCT: randomized controlled trial
